# Effect of Hydroxychloroquine on Lupus Activity, Preeclampsia and Intrauterine Growth Restriction in Pregnant Women with Systemic Lupus Erythematosus and/or Antiphospholipid Syndrome: A Systematic Review and Meta-Analysis

**DOI:** 10.3390/jcm12020485

**Published:** 2023-01-06

**Authors:** Zhengyan Hu, Rui Gao, Wanrong Huang, Huiqing Wang, Lang Qin

**Affiliations:** 1 The Reproductive Medical Center, Department of Obstetrics and Gynecology, West China Second University Hospital, Sichuan University, Chengdu 610041, China; 2 Key Laboratory of Birth Defects and Related of Women and Children of Ministry of Education, West China Second University Hospital, Sichuan University, Chengdu 610041, China; 3 West China School of Medicine, Sichuan University, Chengdu 610041, China; 4 Department of Dermatology, The First Hospital of China Medical University, Shenyang 110001, China; 5 Medical Simulation Centre, West China Second University Hospital, Sichuan University, Chengdu 610041, China

**Keywords:** systematic lupus erythematosus, antiphospholipid syndrome, pregnancy, hydroxychloroquine, preeclampsia, intrauterine growth restriction

## Abstract

Background: Hydroxychloroquine (HCQ) has been used in the treatment of systematic lupus erythematosus (SLE) and antiphospholipid syndrome (APS), but its effect on lupus activity during pregnancy, preeclampsia and intrauterine growth restriction (IUGR) remains unclear. Methods: PubMed, Embase and Cochrane databases were searched before 11 September 2022 for randomized clinical trials (RCT) or observational studies involving additional HCQ treatment and pregnant women diagnosed as having SLE and/or APS/positive antiphospholipid antibodies (aPLs). Risks of high lupus activity, preeclampsia and IUGR were explored. Results: One RCT and 13 cohort studies were included. A total of 1764 pregnancies were included in the pooled meta-analysis (709 in the HCQ group vs. 1055 in the control group). After the additional use of HCQ, the risk of high lupus activity decreased (RR: 0.74, 95% CI: 0.57–0.97, *p* = 0.03). For preeclampsia, the total incidence decreased (RR: 0.54, 95% CI: 0.37–0.78, *p* = 0.001). The subgroup analysis showed statistical significance in the SLE subgroup (RR: 0.51, 95% CI: 0.34–0.78, *p* = 0.002) but not in the APS/aPLs subgroup (RR: 0.66, 95% CI: 0.29–1.54, *p* = 0.34). For IUGR, the decrease in incidence was not statistically significant (RR: 0.80, 95% CI: 0.47–1.35, *p* = 0.46), neither in the SLE subgroup (RR: 0.74, 95% CI: 0.40–1.36, *p* = 0.33) nor in the APS/aPLs subgroup (RR: 1.26, 95% CI: 0.34–4.61, *p* = 0.73). Conclusion: The additional use of HCQ may decrease the risk of high lupus activity during pregnancy and the incidence of preeclampsia for SLE patients, but the results do not support that using HCQ decreases the incidence of preeclampsia for APS/aPLs patients or reduces IUGR risk for SLE and/or APS/aPLs patients.

## 1. Introduction

Systemic lupus erythematosus (SLE) and antiphospholipid syndrome (APS) are two of the most important systemic autoimmune diseases for women of childbearing age. SLE is characterized by the presence of antinuclear antibodies and the formulation of immune complexes, causing inflammation of multiple organs [1]. APS is described as the presence of antiphospholipid antibodies (aPLs) and the clinical symptoms of thrombus or adverse pregnancy outcomes [2]. Primary APS occurs without other autoimmune manifestations, while most instances of APS are associated with SLE and are called secondary APS [2]. Clinical evidence shows that both SLE, APS and positive aPLs are strong risk factors of adverse pregnancy outcomes. Reproductive health is of great importance for woman of childbearing age with SLE and/or APS, which increases the incidence of preterm birth, pregnancy loss, preeclampsia and intrauterine growth restriction (IUGR) [3,4,5] and were regarded as contraindications of pregnancy in the last century. Pregnancy is also a trigger of disease activity for SLE and/or APS. However, in the past few decades, the discovery and use of new drugs have increased the possibility and safety of pregnancy for women with SLE and/or APS.

Hydroxychloroquine (HCQ) is a kind of synthetic 4-aminoquinoline antimalarial drug. Recently, more studies regarding the clinical effect of HCQ—apart from its antimalarial effect–were performed, including anti-inflammatory and immunomodulation, vascular protection and thrombosis prevention, and the regulation of glucose and lipid metabolism [6,7]. The discovery of these effects facilitated the promotion of HCQ from an antimalarial to a rheumatic drug. HCQ is now widely used in the treatment of SLE and is used for pregnant women with SLE and/or APS for its relative safety [8]. However, the detailed benefits of HCQ for pregnant women with SLE and/or APS remain unclear. Considering the complex immunological alterations during pregnancy, it is reasonable to suspect that the efficacy of HCQ for immune-related diseases may differ from that of the general state. Few studies have focused on lupus activity during pregnancy, and the impact of HCQ on pregnancy complications remains controversial.

At present, a few meta-analyses concerning the efficacy of HCQ in pregnancy with SLE or APS have been published [9,10,11]. However, there is still a lack of high-quality evidence on the role of HCQ in lupus activity during pregnancy, preeclampsia and IUGR of patients with SLE and/or APS. This systematic review and meta-analysis focuses on the impact of HCQ on lupus activity in pregnant women with SLE, and pregnancy complications (preeclampsia and IUGR) in pregnant women with SLE and/or APS.

## 2. Methods

This systematic review and meta-analysis was established following the standardized protocol formulated in accordance with the PICO principles, and they were reported according to the Preferred Reporting Items for Systematic Review and Meta-Analysis (PRISMA) guidelines [12]. This study was based on previously published studies and did not contain any new information about human participants or animals.

### 2.1. Literature Search

The literature search strategy was determined by all authors before literature retrieval. A systematic search of the literature was conducted by PubMed (from 1996), Embase (from 1947) and the Cochrane Register of Controlled Trials (from 1996) databases, and all studies published from database establishment to 16 April 2022 were recruited. Search terms including “hydroxychloroquine” and “pregnancy” were used. Language was limited to English. Conference proceedings from 2012 to 2022 were also searched through Embase. Two authors performed the search independently, summarized all retrieved literature and then deleted any duplications. The search strategies are shown in Table S1 of the Electronic Supplementary Material.

### 2.2. Research Selection

We explored the association between the additional use of HCQ during pregnancy and lupus activity, preeclampsia incidence, IUGR incidence. The inclusion and exclusion criteria were determined before study selection. Two authors, independently and step-by-step, scanned the title, abstract and full text to evaluate whether these studies met the inclusion criteria. If there was any dispute, the third author would discuss with them and make decisions until the final results were reached. Studies for this analysis were selected in accordance with the following criteria: (1) study population: pregnant women with SLE, APS or positive aPLs; (2) study design: observational studies or randomized controlled trials (RCTs) that compared the additional use of HCQ in pregnancy with the other group treated without HCQ (including placebo); (3) outcomes: high lupus activity during pregnancy, IUGR, preeclampsia. High lupus activity was defined based on the SLE Disease Activity Index (SLEDAI). IUGR was defined as an estimated fetal weight or abdominal circumference less than the 10th percentile for gestational age. Preeclampsia was defined as hypertension with proteinuria or other end-organ damage after 20 weeks of pregnancy. Specific diagnostic criteria referring to international or local guidelines were acceptable. The standardized review protocol and study eligibility criteria are provided in Table S2.

### 2.3. Data Extraction and Quality Assessment

All the data of interest were independently extracted by two of the authors onto a standardized form. Disagreements were resolved and corrected by consensus. From each study, we collected the study source (lead author, year of publishment), study design (research type, study population, treatment information) and outcomes of interest (high lupus activity during pregnancy, preeclampsia, IUGR).

The quality of all the included studies was independently assessed by two of the authors, and all the disagreements were resolved through discussion. The quality of RCT was evaluated using the Cochrane Collaboration’s tool for the risk of bias [13]. Six domains of bias are included in this instrument, which are selection bias (random sequence generation, allocation concealment), performance bias (blinding of participants and personnel), detection bias (blinding of outcome assessment), attrition bias (incomplete outcome data), reporting bias (selective reporting), and other bias. The quality of observational cohort studies was assessed using the Newcastle–Ottawa Scale (NOS) [14]. Three categories of bias (selection, comparability and outcome) are evaluated and scored by answering 8 questions, with a maximum of 9 points.

### 2.4. Statistical Analysis

Data for all the dichotomous outcomes were pooled and analyzed by Cochrane Collaboration Review Manager 5.4.1 software (Nordic Cochrane Center). Comparisons were conducted between the HCQ group and control group (placebo or treatment without HCQ). We performed a standard pairwise meta-analysis for each dichotomous outcome, with the calculation of relative risk (RR) and 95% confidence intervals (CI).

Subgroup analyses were conducted for any differences in the effects of HCQ based on the following factors: study design (RCT or observational study), diagnostic criteria for high lupus activity, and study population (SLE patients with/without aPLs or primary/secondary APS/aPLs patients).

Heterogeneity between studies was estimated by I^2^ statistic and a Cochran Q test. If I^2^ ≥ 50% or *p* < 0.1, heterogeneity was considered high and a random-effect model was used; if I^2^ < 50% or *p* ≥ 0.1, heterogeneity was accepted and a fixed-effect model was used. When considerable heterogeneity appeared, a sensitivity analysis was performed by changing the analysis model or reviewed articles one-by-one to confirm the cause of heterogeneity. If equal to or more than 10 studies were included in a group or a subgroup, funnel plots were made to assess the publication bias. The symmetrical distribution of points on the funnel plot indicated no bias or small bias.

## 3. Results

**Figure 1:**
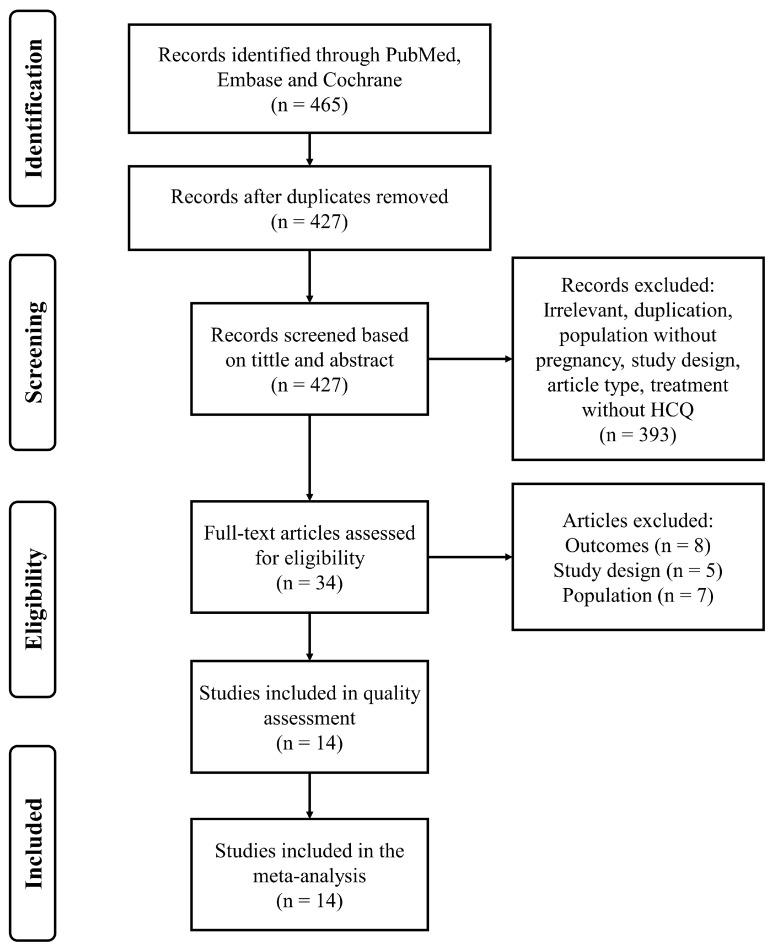
PRISMA flow chart.

A total of 465 records were identified during the search of PubMed (60), Embase (400) and Cochrane (5); 427 records were kept after removing duplicates. Three hundred and ninety-three records were excluded on the basis of title and abstract, mainly because of irrelevant research and study population. Based on full-text screening of the 34 studies, another 20 studies were excluded, mainly for lack of appropriate comparison/treatment group and lack of outcomes of interest. Fourteen studies met the inclusion criteria and were accepted for data extraction and quality assessment. The PRISMA flow chart is shown in Figure 1.

This systematic review and meta-analysis took in one RCT and 13 observational cohort studies. The publication date ranged from 2001 to 2022. Research populations included SLE (11 studies) and APS or positive aPLs (three studies). A total of 1856 pregnancies were involved, with 1764 included in the pooled meta-analysis (709 in the HCQ group vs. 1055 in the control group). The characteristics of the 14 studies and the available data of each study are presented in Table S5.

One RCT [15] was included in the Cochrane risk of bias assessment. The evaluation result is presented in Table S3. The risk of bias was generally low, but selection bias (random sequence generation and allocation concealment) and detection bias (blinding of outcomes assessment) were regarded as unclear, as a detailed description was not provided.

Thirteen observational studies [16,17,18,19,20,21,22,23,24,25,26,27,28] were included in the NOS quality assessment. A summary of the evaluation result is presented in Table S4. Seven studies were scored as eight points and six studies were scored as nine. All the studies were scored as the maximum of four points in the selection category, indicating that both the exposed cohort and non-exposed cohort in each study were representative. For the comparability category, we determined pregnancy as the most important factor and diseases condition as the second. Seven studies [16,17,18,19,23,26,27] were scored as one point for not controlling for or not mentioning the second important factor, which means there may be differences in diseases condition between the two cohorts. The other six studies [20,21,22,24,25,28] were scored as the maximum of two points. Finally, for the outcome category, all the studies were scored as the maximum of three points, indicating that the outcome data were reliable.

As for background medication, many patients with SLE received corticosteroid, and some received azathioprine, aspirin and/or heparin. All the patients with APS/aPLs received low-dose aspirin (LDA) and low-molecular-weight heparin (LMWH).

A summarization of all the results is presented in Table 1.

### 3.1. High Lupus Activity during Pregnancy

**Figure 2:**
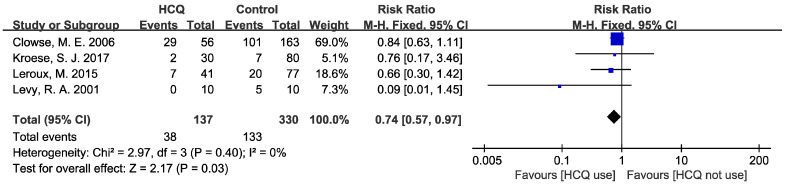
Forest plot for RR of high lupus activity. The pooled estimate of RR of high lupus activity was determined for patients taking HCQ compared to placebo or those not taking HCQ.

The result of the pooled meta-analysis for high lupus activity during pregnancy is presented in Figure 2. Four studies [15,18,20,22] reported lupus activity or lupus flare, containing one RCT and three cohorts. The SLEDAI was used to describe lupus activity in all four studies. A SLEDAI-based lupus flare (an increase in ≥3 scores) or a high SLEDAI score ≥ 4 is accepted as a description for high lupus activity. The HCQ group took in 137 pregnancies and the control group took in 330 pregnancies. Compared to the control group, the risk of high lupus activity was reduced by 26% when HCQ was used, and this result was statistically significant (RR: 0.74, 95% CI: 0.57–0.97, *p* = 0.03). There was no statistical heterogeneity among these four studies (I^2^ = 0%, *p* = 0.40), so a fixed-effect model was used to calculate RR and 95% CI. Considering the differences in the study design and diagnostic criteria for high lupus activity, sensitivity analyses removing the only RCT or the only study with different diagnostic criteria were conducted. However, neither of the analyses revealed significant results, as shown in Table 2. Moreover, one of these studies [18] reported an increase in lupus activity and lupus flares during pregnancy in patients who had stopped taking HCQ, compared to either the continuous-HCQ-use cohort or the non-exposed cohort. A summarization of detailed data is shown in Table S6. Publication bias was not assessed because the number of included studies was less than 10.

### 3.2. Intrauterine Growth Restriction

**Figure 3:**
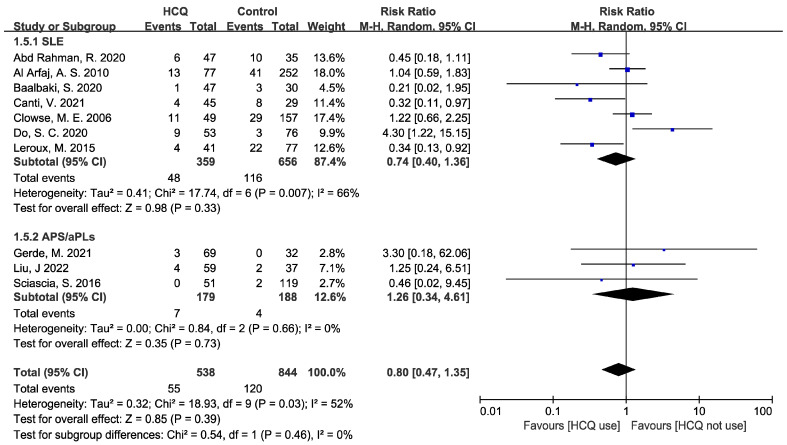
Forest plot for RR of IUGR. The pooled estimate of the RR of IUGR was determined for patients taking HCQ compared to those not taking HCQ. A subgroup analysis was conducted based on the study population.

Ten cohort studies [16,18,21,22,23,24,25,26,27,28] reported IUGR incidence, with seven concerning SLE and three concerning APS/aPLs. A summarization of detailed data is presented in Table S7. The HCQ group took in 538 pregnancies and the control group took in 844 pregnancies. Overall, the risk of IUGR in the HCQ group was reduced by 20% compared to the control group, but the result was not statistically significant (RR: 0.80, 95% CI: 0.47–1.35, *p* = 0.46), as shown in Figure 3. The heterogeneity was moderate (I^2^ = 52%, *p* = 0.03) and the random-effect model was used to calculate RR and 95% CI. A subgroup analysis was conducted based on the study population. In the SLE subgroup, compared to the control group (n = 656), the risk of IUGR was reduced by 26% when using HCQ additionally (n = 359), and this result was not statistically significant (RR: 0.74, 95% CI: 0.40–1.36, *p* = 0.33). The heterogeneity was relatively high (I^2^ = 66%, *p* = 0.007). A sensitivity analysis was performed by removing the study one by one and analyzing the others to estimate whether the result could be remarkably affected by a single study; the result of the sensitivity analysis is presented in Table S8. During the sensitivity analysis, one study [16] had a large effect on the results of the meta-analysis. After removing the study, a statistically significant result was obtained (RR: 0.70, 95% CI: 0.50–0.96, *p* = 0.03), while the heterogeneity remained moderate (I^2^ = 54%, *p* = 0.05). This effect may be attributed to the different baseline characteristics of disease states in the study populations and potential publication bias. However, in the APS subgroup, compared to the control group (n = 188), the risk of IUGR increased by 26% when using HCQ additionally (n = 179), but this result was not statistically significant (RR: 1.26, 95% CI: 0.34–4.61, *p* = 0.73). There was no heterogeneity among these three studies (I^2^ = 0%, *p* = 0.84). Publication bias was not assessed because the number of included studies was less than 10 in each subgroup.

### 3.3. Preeclampsia

**Figure 4:**
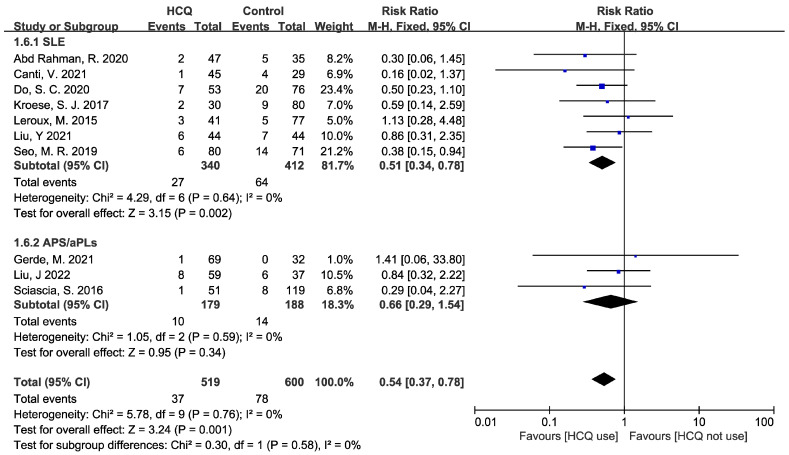
Forest plot for the RR of preeclampsia. The pooled estimate of the RR of preeclampsia was determined for patients taking HCQ compared to those not taking HCQ. A subgroup analysis was conducted based on the study population.

Ten cohort studies [16,17,19,20,21,22,23,24,25,27] reported preeclampsia incidence, including seven SLE studies and three APS/aPLs studies. A summarization of detailed data is presented in Table S9. The HCQ group took in 519 pregnancies and the control group took in 600 pregnancies. As is shown in Figure 4, compared to the control group, the total risk of preeclampsia was reduced by 46% when using HCQ additionally, and this result was statistically significant (RR: 0.54, 95% CI: 0.37–0.78, *p* = 0.001). There was no heterogeneity among the 10 studies (I^2^ = 0%, *p* = 0.76), so a fixed-effect model was used to calculate the RR and 95% CI. A subgroup analysis was conducted based on the study population. In the SLE subgroup, compared to the control group (n = 412), the risk of preeclampsia was reduced by 49% in the HCQ group (n = 340), and this result was statistically significant (RR: 0.51, 95% CI: 0.34–0.78, *p* = 0.002) without heterogeneity (I^2^ = 0%, *p* = 0.64). On the other hand, in the APS/aPLs subgroup, the risk of preeclampsia reduced by 34% in the HCQ group (n = 179) compared to the control group (n=188), but this result was not statistically significant (RR: 0.66, 95% CI: 0.29–1.54, *p* = 0.34) without heterogeneity (I^2^ = 0%, *p* = 0.59). Publication bias was not assessed because the number of included studies was less than 10 in each subgroup.

## 4. Discussion

This systematic review and meta-analysis was performed to provide clinical evidence on the effects of the additional use of HCQ on lupus activity, IUGR incidence and preeclampsia incidence in pregnant women with SLE and/or APS/aPLs. Our results suggested that the additional use of HCQ may decrease the risk of high lupus activity during pregnancy and decrease the incidence of preeclampsia in SLE patients, but not in APS/aPLs patients. However, HCQ does not seem to decrease the incidence of IUGR, either in SLE patients or in APS/aPLs patients.

The efficacy of HCQ in preventing lupus flare has been well researched in the general patient population [29], but studies for pregnant women are scant, especially high-quality RCTs or cohorts. Therefore, meta-analysis is lacking. As far as we know, this is the first study to synthesize published evidence regarding the effect of additional HCQ use in controlling lupus activity during pregnancy. Our data suggest that continuous additional HCQ use in pregnancy can decrease the risk of high lupus activity. However, during the sensitivity analyses, the results did not reveal such a reduction. In addition, the small number of studies could be the reason for this result. Therefore, more high-quality studies focused on this outcome should be performed. Moreover, although a study reported adverse effects when discontinuing HCQ during pregnancy, no definitive conclusion can be drawn from the small number of studies.

In terms of pregnancy complications, the results of previous SLE studies are controversial and the number of APS/aPLs studies is limited. In addition, no studies have pooled and compared SLE and APS studies. Our results differ between preeclampsia and IUGR, and between SLE and APS/aPLs patients. The effects of HCQ on preeclampsia and IUGR should be related to the interaction of their mechanism. According to previous research, HCQ can play a role in immunomodulation, endothelial protection and metabolic regulation through multiple pathways, thus, protecting placental function and reducing the incidence of placenta-mediated complications. As for immunomodulation and anti-inflammation, HCQ may affect innate immunity by inhibiting toll-like receptors (TLR)-3, -7, and -9, inhibiting the immune response to auto-antigenic peptides by preventing the formulation of peptide-MHC II complex, and maintaining helper T cell (Th)1/Th2 balance by reducing the production of proinflammatory cytokines [30]. In addition, HCQ can provide an alkaline environment to stabilize the lysosomal membrane and inhibit chemotaxis, phagocytosis and antigen presentation [7]. As for endothelial protection, HCQ may prevent endothelial dysfunction by inhibiting the expression of vascular cellular adhesion molecule-1 (VCAM) through the upregulation of ERK5 kinase activity [31]. It may also inhibit the aggregation of platelets and the release of arachidonic acid from activated platelets [32]. Moreover, it was reported that HCQ could decrease aPL levels in plasma and interfere with APS-related pathways [30], preventing thrombogenesis. As for metabolic regulation, according to a meta-analysis [33], HCQ can improve the lipid profile and lower the incidence of diabetes with the molecular pathways being explored.

Preeclampsia and IUGR share several similar pathological processes but still have some differences. Accelerated endothelial dysfunction, hypoxia of the placenta and excessive inflammation up-regulation occur in both preeclampsia and IUGR. However, it seems that higher levels of endothelial activators such as VCAM and intercellular adhesion molecule-1 (ICAM), more significant changes of placenta-derived growth factor (PlGF), and more developed inflammatory states occur in preeclampsia than in IUGR. Moreover, metabolic abnormalities seem to play a larger role in preeclampsia than in IUGR [34]. These differences may lead to different responses to drugs.

Patients with SLE and APS also have similarities and differences. Thrombocytopenia and activation of the complement system happen in both SLE and APS patients [35,36]. In SLE patients, symptoms and complications are often attributed to local tissue inflammatory injury caused by immune complexes [37], and specific risk factors of pregnancy complications include lupus flare during pregnancy and lupus nephritis [35]. In APS patients, thrombogenesis and placental dysfunction (trophoblast damage) caused by aPLs [38] are important in pregnancy complications [36]. Theoretically, the pharmacological effects of HCQ interact with the molecular mechanisms of both SLE and APS. However, different basal treatments of the two diseases may lead to different effects. Yet, without statistical differences, it seems that SLE patients tend to benefit more from the additional use of HCQ in preventing pregnancy complications than APS/aPLs patients. This may be related to the fact that standard treatment for APS (LDA + LMWH) is effective enough for most patients, while the effect of basal treatment for SLE (glucocorticoid) is limited. However, due to the small number of studies on APS and HCQ, no definite conclusions can be drawn. These results agree with the EULAR guidelines and the American College of Rheumatology (ACR) guideline that patients with SLE are recommended to receive HCQ preconceptionally and throughout pregnancy, while only patients with refractory APS may consider HCQ in addition to standard treatment [8,39,40,41].

This systematic review and meta-analysis has some limitations. First, some results may be limited by the small number of studies. Publication bias was not assessed because the number of studies was never more than 10 in each group/subgroup. Second, although we conducted sensitivity analyses and identified some factors, some potential sources of heterogeneity remain to be investigated. Third, due to a lack of data in some original studies, we did not focus on the usage and dosage of HCQ and disease status of the population (refractory/non-refractory APS, SLE with/without lupus nephritis). Fourth, patients with SLE received different basal treatments, and some of them received aspirin, which proved helpful to prevent preeclampsia. Though the characteristics between the HCQ group and the control group in each original study were comparable, there might be potential differences.

## 5. Conclusions

In conclusion, the additional use of HCQ may be beneficial in preventing lupus flare or high lupus activity during pregnancy for patients with SLE. HCQ is associated with a lower risk of preeclampsia in SLE patients, but we did not observe this effect in APS/aPLs patients receiving standard treatment. We failed to prove the efficacy of HCQ in the prevention of IUGR in either SLE or APS/aPLs patients. Our results suggest that HCQ should be conventionally used in pregnancies with SLE, but additional use in pregnancies with APS/aPL without other complications is not currently indicated.

## Figures and Tables

**Table 1:** Relative risk of each outcome.

Outcomes		Number of Studies	Number of Pregnancies (HCQ vs. Control)	RR [95% CI] ^1^	*p* Value	I^2^
High lupus activity		4	137 vs. 330	0.74 [0.57, 0.97]	0.03	0%
IUGR		10	538 vs. 844	0.80 [0.47, 1.35]	0.46	52%
	SLE	7	359 vs. 656	0.74 [0.40, 1.36]	0.33	66%
	APS/aPLs	3	179 vs. 188	1.26 [0.34, 4.61]	0.73	0%
Preeclampsia		10	519 vs. 600	0.54 [0.37, 0.78]	0.001	0%
	SLE	7	340 vs. 412	0.51 [0.34, 0.78]	0.002	0%
	APS/aPLs	3	179 vs. 188	0.66 [0.29, 1.54]	0.34	0%

^1^ Fixed-effect model was used if total I^2^ < 50% and random-effect model was used if total I^2^ > 50%. HCQ: hydroxychloroquine; IUGR: intrauterine growth restriction; SLE: systemic lupus erythematosus; APS: antiphospholipid syndrome; aPLs: antiphospholipid antibodies.

**Table 2:** Sensitivity analyses for the risk of high lupus activity.

Removed Study	No. of Studies	No. of Pregnancies (HCQ vs. Control)	RR [95% CI] *	*p* Value	I^2^
Levy, R. A. 2001 ^1^	3	127 vs. 320	0.80 [0.61, 1.04]	0.10	0%
Clowse, M. E. 2006 ^2^	3	81 vs. 167	0.54 [0.28, 1.03]	0.06	2%

* Fixed-effect model was used. ^1^ The only RCT; ^2^ the only study with different diagnostic criteria.

## Data Availability

All data relevant to the study are available in the article and its online supplementary materials.

## Supplementary Materials

The following supporting information can be downloaded at: https://www.mdpi.com/article/10.3390/jcm12020485/s1, Table S1: Search strategies; Table S2: Standardized review protocol and study eligibility criteria based on the PICO framework; Table S3: Assessment of bias risk using Cochrane Collaboration’s tool; Table S4: Quality assessment of cohort/case-control studies using the Newcastle–Ottawa Scale; Table S5: Study characteristics and availability; Table S6: High lupus activity during pregnancy; Table S7: IUGR; Table S8: Result of sensitivity analysis for IUGR in SLE subgroup; Table S9: Preeclampsia.
