# Supplementary material for: Effect of Hydroxychloroquine on Lupus Activity, Preeclampsia and Intrauterine Growth Restriction in Pregnant Women with Systemic Lupus Erythematosus and/or Antiphospholipid Syndrome: A Systematic Review and Meta-Analysis
Source: J Clin Med. 2023 Jan 6;12(2):485. doi: 10.3390/jcm12020485 (PMC9866542; doi:10.3390/jcm12020485)
Supplement: Supplementary file 1 [file jcm-12-00485-s001.zip › jcm-2076590-supplementary.pdf]

Table S1: Search strategies

**Database: PubMed**	
**#**	**Search date: Sept 11, 2022**
1	("Hydroxychloroquine"[Mesh] OR Hydroxychloroquine[tiab] OR Sinonteam[tiab] OR Woodcock[tiab] OR Plaquenil[tiab] OR Quineprox[tiab])
2	("Pregnancy"[Mesh] OR pregnan*[tiab])
3	("lupus erythematosus, systemic"[Mesh] OR "antiphospholipid syndrome"[Mesh] OR "antibodies, antiphospholipid"[Mesh])
4	1 AND 2 AND 3
5	(animals[mh] NOT humans[mh])
6	4 NOT 5
7	(clinical trial[pt] OR comparative study[pt] OR multicenter study[pt] OR validation studies[pt] OR cohort studies[mh] OR cross-over studies[mh] OR case-control studies[mh] OR follow-up studies[mh] OR cross-sectional studies[mh] OR (case reports[pt] AND series[tiab]))
8	6 AND 7
9	(review[pt] OR review[ti] OR comment[pt] OR editorial[pt] OR meta-analysis[pt] OR meta-analysis[ti] OR letter[pt] OR in vitro techniques[mh] OR news[pt] OR guideline[pt])
10	8 NOT 9
11	10 AND eng[la]
**Database: Embase**	
**#**	**Search date: Sept 11, 2022**<br>**Segment used: 1974 to Sept 11, 2022**
1	*Hydroxychloroquine/ or (Hydroxychloroquine or Sinonteam or Woodcock or Plaquenil or Quineprox).ti,ab.
2	exp pregnancy/ or (pregnan$).ti,ab.
3	1 and 2
4	exp systemic lupus erythematosus
5	exp antiphospholipid syndrome
6	exp antiphospholipid antibodies
7	4 or 5 or 6
8	3 and 7
9	limit 8 to english language
10	9 not ((exp animal/ or nonhuman/) not exp human/)
11	limit 10 to (yr="2012 -Current" and (conference abstract or conference proceeding or "conference review"))

12	limit 10 to (article or conference paper)
13	11 or 12
14	remove duplicates from 13
**Database: Cochrane Central Register of Controlled Trials (CENTRAL)**	
**#**	**Search date: Sept 11, 2022**
1	[mh "Hydroxychloroquine"] or Hydroxychloroquine or Sinonteam or Woodcock or Plaquenil or Quineprox:ti,ab,kw
2	[mh "Pregnancy"]or pregnan*:ti,ab,kw
3	[mh "systemic lupus erythematosus"] or [mh "antiphospholipid syndrome"] or [mh "antiphospholipid antibodies"]
4	#1 and #2 and #3
5	#4 not (pubmed or embase):an

Table S2: Standardized review protocol and study eligibility criteria based on the PICO framework

Category	Inclusion criteria
**Population**	• Pregnant women with systemic lupus erythematosus, antiphospholipid syndrome or positive antiphospholipid antibodies
**Intervention**	• Hydroxychloroquine
**Comparison**	• Any
**Outcome**	• High lupus activity (for SLE patients)<br>• Preeclampsia<br>• Intrauterine growth restriction
**Study type**	• Comparative studies (clinical trials and observational studies)<br>• <u>*Exclude:*</u><br>Non-comparative studies
**Language**	• English
**Publication date limits**	• **Databases:** From inception to Sept 11, 2022<br>• **Conferences Proceedings:** Past 10 years from 2012 to 2022

Table S3: Assessment of bias risk using Cochrane Collaboration's Tool

Study	Selection bias		Performance bias	Detection bias	Attrition bias	Reporting bias	Other bias
	**Random sequence generation**	**Allocation concealment**	**Blinding of participants and personnel**	**Blinding of outcome assessment**	**Incomplete outcome data**	**Selective reporting**	**Other sources of bias**
Levy, R. A. 2001	Unclear risk	Unclear risk	Low risk	Unclear risk	Low risk	Low risk	Low risk

Table S4: Quality assessment of cohort/case-control studies using the Newcastle-Ottawa Scale

Study	Study design	Selection				Comparability	Outcome			Final score
		representativeness of the exposed cohort	selection of the non-exposed cohort	ascertainment of exposure	outcome of interest was not present at start of study	comparability of cohorts on the basis of the design or analysis	assessment of outcome	follow-up long enough for outcomes to occur	adequacy of follow up of cohorts	
Clowse, M. E. 2006	Cohort	*	*	*	*	*	*	*	*	8
Al Arfaj, A. S. 2010	Cohort	*	*	*	*	*	*	*	*	8
Leroux, M. 2015	Cohort	*	*	*	*	**	*	*	*	9
Sciascia, S. 2016	Cohort	*	*	*	*	*	*	*	*	8
Kroese, S. J. 2017	Cohort	*	*	*	*	**	*	*	*	9
Seo, M. R. 2019	Cohort	*	*	*	*	*	*	*	*	8
Abd Rahman, R. 2020	Cohort	*	*	*	*	**	*	*	*	9
Baalbaki, S. 2020	Cohort	*	*	*	*	**	*	*	*	9
Do, S. C. 2020	Cohort	*	*	*	*	*	*	*	*	8
Canti, V. 2021	Cohort	*	*	*	*	**	*	*	*	9
Gerde, M. 2021	Cohort	*	*	*	*	**	*	*	*	9
Liu, Y 2021	Cohort	*	*	*	*	*	*	*	*	8
Liu, J 2022	Cohort	*	*	*	*	*	*	*	*	8

Table S5: Study characteristics and availability

	Research type	Number of pregnancies	Study population	Treatment/Comparator Groups (n)	Outcomes reported
Levy, R. A. 2001	RCT, single center	20	SLE, DLE	a) HCQ (10)<br>b) placebo (10)	lupus activity
Clowse, M. E. 2006	Prospective cohort, single center	257	SLE	a) No HCQ (163)<br>b) HCQ (56)<br>c) HCQ stopped (38)	lupus activity, IUGR
Al Arfaj, A. S. 2010	Retrospective study, single center	383	SLE	a) Prednisolone (222)<br>b) Prednisolone + HCQ (69)<br>c) Prednisolone + azathioprine (30)<br>d) Prednisolone + azathioprine + HCQ (8)<br>e) None (54)	IUGR
Leroux, M. 2015	Retrospective cohort, single center	118	SLE	a) HCQ (41)<br>b) no HCQ (77)	lupus activity, IUGR, preeclampsia

Sciascia, S. 2016	Retrospective cohort, single center	170	aPLs	a) HCQ (51)<br>b) no HCQ (119)	preeclampsia, IUGR
Kroese, S. J. 2017	Retrospective cohort, single center	110	SLE	a) HCQ (30)<br>b) no HCQ (80)	lupus activity, preeclampsia
Seo, M. R. 2019	Retrospective cohort, single center	151	SLE	a) HCQ (80)<br>b) no HCQ (71)	preeclampsia
Abd Rahman, R. 2020	Retrospective cohort, single center	82	SLE	a) HCQ (47)<br>b) no HCQ (35)	IUGR, preeclampsia
Baalbaki, S. 2020	Retrospective cohort, single center	77	SLE	a) HCQ (47)<br>b) no HCQ (30)	IUGR
Do, S. C. 2020	Retrospective cohort, single center	129	SLE	a) HCQ (53)<br>b) no HCQ (76)	preeclampsia, IUGR
Canti, V. 2021	Prospective cohort, single center	74	SLE	a) HCQ (45)<br>b) no HCQ (29)	preeclampsia, IUGR
Gerde, M. 2021	Retrospective cohort, single center	101	APS	a) HCQ + LDA + LMWH (69)<br>b) LDA + LMWH (32)	IUGR, preeclampsia
Liu, Y 2021	Retrospective cohort, single center	88	SLE	a) HCQ (44)<br>b) no HCQ (44)	preeclampsia
Liu, J 2022	Retrospective cohort, single center	96	APS	a) HCQ (59)<br>b) no HCQ (37)	IUGR, preeclampsia

SLE: systemic lupus erythematosus; DLE: discoid lupus erythematosus; HCQ: Hydroxychloroquine; APS: antiphospholipid syndrome; aPLs: antiphospholipid antibodies; IUGR: intrauterine growth restriction

Table S6: High lupus activity during pregnancy

	**HCQ (n/N)**	**Comparison (n/N)**	**Criterion**
Levy, R. A. 2001	HCQ: 0/10	Placebo: 5/10	SLEDAI≥4
Clowse, M. E. 2006	HCQ: 29/56	no HCQ: 101/163<br>HCQ stopped: 32/38	SLEDAI increase ≥3
Leroux, M. 2015	HCQ: 7/41	no HCQ: 20/77	SLEDAI≥4
Kroese, S. J. 2017	HCQ: 2/30	no HCQ: 7/80	SLEDAI≥4
**Overall**	**HCQ: 38/137**	**no HCQ: 133/330**	

Table S7: IUGR

	**HCQ (n/N)**	**Comparison (n/N)**
**SLE subgroup**	**HCQ: 48/359**	**no HCQ: 116/656**
Clowse, M. E. 2006	HCQ: 11/49	no HCQ: 29/157<br>HCQ stopped: 7/34
Leroux, M. 2015	HCQ: 4/41	no HCQ: 22/77
Al Arfaj, A. S. 2010	HCQ: 13/77	no HCQ: 41/252
Abd Rahman, R. 2020	HCQ: 6/47	no HCQ: 10/35
Baalbaki, S. 2020	HCQ: 1/47	no HCQ: 3/30
Do, S. C. 2020	HCQ: 9/53	no HCQ: 3/76
Canti, V. 2021	HCQ: 4/45	no HCQ: 8/29

APS/aPLs subgroup	HCQ: 7/179	no HCQ: 4/188
Sciascia, S. 2016	HCQ: 0/51	no HCQ: 2/119
Gerde, M. 2021	HCQ: 3/69	no HCQ: 0/32
Liu, J 2022	HCQ: 4/59	no HCQ: 2/37
**Overall**	**HCQ: 55/538**	**no HCQ: 120/844**

Table S8: Result of sensitivity analysis for IUGR in SLE subgroup

Removed study	No. of pregnancies (HCQ vs Control)	RR (95% CI) [a]	I^2
Clowse, M. E. 2006	310 vs 499	0.72 [0.51, 1.03]	68%
Leroux, M. 2015	318 vs 579	0.93 [0.68, 1.29]	65%
Abd Rahman, R. 2020	312 vs 621	0.88 [0.64, 1.22]	68%
Al Arfaj, A. S. 2010	282 vs 404	0.74 [0.51, 1.06]	70%
Baalbaki, S. 2020	312 vs 626	0.84 [0.62, 1.15]	69%
Do, S. C. 2020	306 vs 580	0.70 [0.50, 0.96]	54%
Canti, V. 2021	314 vs 627	0.89 [0.64, 1.22]	66%

[a] RR was calculated using fixed-effect model in sensitivity analysis

Table S9: Preeclampsia

	HCQ (n/N)	Comparison (n/N)
**SLE subgroup**	**HCQ: 27/340**	**no HCQ: 64/412**
Leroux, M. 2015	HCQ: 3/41	no HCQ: 5/77
Kroese, S. J. 2017	HCQ: 2/30	no HCQ: 9/80
Seo, M. R. 2019	HCQ: 6/80	no HCQ: 14/71
Abd Rahman, R. 2020	HCQ: 2/47	no HCQ: 5/35
Do, S. C. 2020	HCQ: 7/53	no HCQ: 20/76
Canti, V. 2021	HCQ: 1/45	no HCQ: 4/29
Liu, Y 2021	HCQ: 6/44	no HCQ: 7/44
**APS/aPLs subgroup**	**HCQ: 10/179**	**no HCQ: 14/188**
Sciascia, S. 2016	HCQ: 1/51	no HCQ: 8/119
Gerde, M. 2021	HCQ: 1/69	no HCQ: 0/32
Liu, J 2022	HCQ: 8/59	no HCQ: 6/37
**Overall**	**HCQ: 37/519**	**no HCQ: 78/600**
